# Transcriptomic and microRNA Profiling Suggest a Candidate RIPPLY1–cfa‐miR‐187 Relationship in Canine Mammary Gland Tumours From Dogs With Confirmed Metastasis

**DOI:** 10.1111/vco.70084

**Published:** 2026-07-03

**Authors:** Shaohsu Wang, Masashi Takahashi, Hui‐Wen Chen, Nobuhiro Nozaki, Mohammad Arif, Yutaro Ide, Yoshiyuki Akiyama, Sirazul Islam, Tatsuro Hifumi, Shinji Hirano, Noriaki Miyoshi, Osamu Yamato, Daiki Kato, Takayuki Nakagawa, Naoki Miura

**Affiliations:** ^1^ Joint Graduate School of Veterinary Medicine Kagoshima University Kagoshima Shi Kagoshima Ken Japan; ^2^ Veterinary Teaching Hospital, Joint Faculty of Veterinary Medicine Kagoshima University Kagoshima Shi Kagoshima Ken Japan; ^3^ Department of Microbiology and Hygiene Bangladesh Agricultural University Mymensingh Bangladesh; ^4^ Department of Livestock Services (DLS) Ministry of Fisheries and Livestock Dhaka Bangladesh; ^5^ Laboratory of Veterinary Surgery, Graduate School of Agricultural and Life Sciences The University of Tokyo Tokyo Japan

**Keywords:** candidate biomarker, coding sequence binding, comparative oncology, dog, miRNA–mRNA interaction

## Abstract

Canine mammary gland tumours (MGT) represent a common malignancy in intact female dogs, with a high metastasis rate approaching 50% and a poor prognosis. However, metastasis‐related genes remain largely unelucidated. Accordingly, we aimed to identify differentially expressed mRNAs and miRNAs in primary canine MGT tissues from dogs with or without confirmed metastasis. We compared mRNA and microRNA (miRNA) expression profiles between malignant MGT tissue samples with and without metastasis. Differentially expressed genes and miRNAs were identified using next‐generation sequencing and analysed using the Empirical Analysis of DEGs tool within CLC Genomics Workbench. We identified 119 genes and eight miRNAs as differentially expressed in primary MGTs from dogs with confirmed metastasis. The RIPPLY1 gene was significantly downregulated in primary MGT samples with confirmed metastasis and this result was validated by RT‐qPCR. The Cancer Genome Atlas (TCGA) database showed decreased RIPPLY1 expression in human breast cancer, supporting its possible relevance to tumour biology. In silico analysis (miRWalk) revealed that miR‐187 is a predicted candidate miRNA that may interact with RIPPLY1 and both NGS data and RT‐qPCR showed increased cfa‐miR‐187 expression in primary tumour samples from dogs with confirmed metastasis. The inverse expression pattern of RIPPLY1 and cfa‐miR‐187, together with RNAhybrid binding prediction, suggests a possible candidate miRNA–mRNA relationship. STRING analysis suggested a possible pathway context involving beta‐catenin/TCF‐related proteins, but this computational result requires further validation. Based on these findings, we identified RIPPLY1 and cfa‐miR‐187 as potential candidate transcripts associated with primary canine MGTs from dogs with confirmed metastasis but functional validation is required.

## 1 Introduction

Canine mammary gland tumours (MGTs) are common tumours in intact female dogs, and malignant canine MGTs are associated with a poor prognosis with metastasis occurring in approximately 50% of cases [1, 2]. Previous studies have shown that malignant canine MGTs with lymphatic invasion or advanced clinical stage are associated with poorer prognosis [2, 3]. Therefore, earlier diagnosis is key to improving outcomes for canine MGTs and would be greatly aided by establishing reliable biomarkers.

Veterinary oncologists have investigated a number of proposed diagnostic biomarkers for canine MGTs in recent years and these include crystallin alphaB (CRYAB), carcinoembryonic antigen (CEA), cancer antigen 15‐3 (CA15‐3) and Ki‐67. Such biomarkers have largely been assessed for potential clinical utility as an indicator of tumour aggressiveness [4]; however, they are largely derived from human breast cancer studies and their applicability in veterinary medicine is still limited [5].

MicroRNAs (miRNAs) represent another promising area for biomarker research. They are short non‐coding RNAs, normally 18–28 nucleotides in length and regulate gene expression post‐transcriptionally by promoting mRNA degradation or inhibiting translation. Several studies have shown the involvement of miRNAs in cancer development and progression, including in canine MGTs [5, 6, 7, 8, 9]. Studies on expression of miRNAs and the RNAs they target thus have the potential to identify potential biomarkers and throw light on mechanisms underlying tumourigenesis and metastasis. Expression of the relevant genes can be comprehensively profiled through next‐generation sequencing (NGS), which facilitates the identification of novel molecular signatures for cancer types [10]. We have previously reported on NGS‐based investigations of miRNA expression in veterinary cancers [11], but the issue of concomitantly differentially expressed genes and miRNAs in metastatic malignant canine MGTs has remained largely unaddressed.

Previous studies have investigated the transcriptomic profile in metastatic and non‐metastatic canine tumours, highlighting the complex nature of the metastatic transcriptome. miRNA–mRNA relationships may contribute to cancer progression and may provide candidate biomarkers [12, 13]. For example, the interaction of miR‐200 and PGI/AMF is a key regulator of human breast cancer metastasis by inducing epithelial‐mesenchymal transition [14]. Therefore, paired mRNA and miRNA profiling may help identify candidate molecular relationships associated with aggressive tumour behaviour in canine MGTs.

Accordingly, in this study, we aimed to identify differentially expressed mRNAs and miRNAs associated with canine primary MGTs with confirmed metastasis using transcriptome profiling and to explore a candidate miRNA–mRNA relationship among the prioritised transcripts for the generation of a hypothesis on molecular markers associated with canine primary MGTs with confirmed metastasis.

## 2 Materials and Methods

### 2.1 Clinical Samples and Cell Lines

We targeted 23 tissue samples that had previously been collected from female dogs diagnosed with metastatic (*n* = 10) or non‐metastatic (*n* = 13) MGTs at the Kagoshima University Veterinary Teaching Hospital (KUVTH) or an affiliated animal hospital. A diagnosis of MGT had been made in each case by three certified veterinary pathologists (T.H., S. H. and N. M.) affiliated with our institution, through examination of haematoxylin‐and‐eosin‐stained specimens, based on published standards for the classification and grading of canine MGTs [15, 16]. Cases were regarded as metastasized when remote tumours were identified in lymph nodes and/or other organs. However, all clinical tissue samples submitted for RNA extraction in this study were collected from primary MGTs. These primary tumour tissues were processed as bulk samples without prior microdissection or cell sorting. Therefore, the extracted RNA represented a mixed expression profile derived from neoplastic epithelial cells and non‐neoplastic components of the tumour microenvironment, including stromal and immune cells. Every donor had also received ultrasound and x‐ray examinations and the pathologist made reference to the results of these examinations in making diagnoses. Details on the sample donors are provided in Table 1.

**TABLE 1:** Clinical information on canine MGT tissue.

Diagnosis	Breed	Age (year)	Neutered (Y/N)	Metastasis (M/NM)
Carcinoma	American Cocker Spaniel	6	Y	NM
Carcinoma	Chihuahua	12	Y	NM
Carcinoma	French bulldog	9	N	NM
Carcinoma	Labrador Retriever	14	Y	NM
Carcinoma	Papillon	11	N	NM
Carcinoma	Shiba Inu	10	N	NM
Carcinoma	Welsh Corgi	16	Y	NM
Complex carcinoma	Chihuahua	11	Y	NM
Complex carcinoma	Labrador Retriever	11	Y	NM
Complex carcinoma	Mixed	9	N	NM
Tubulopapillary carcinoma	Chihuahua	17	N	NM
Tubulopapillary carcinoma	Shih Tzu	14	N	NM
Carcinosarcoma	English Springer Spaniel	12	N	NM
Carcinoma	Miniature Schnauzer	11	Y	M
Carcinoma	Mixed	Unknown	Y	M
Carcinosarcoma	Miniature Dachshund	13	N	M
Complex carcinoma	Miniature Dachshund	13	Y	M
Osteosarcoma	Beagle	12	N	M
Tubulopapillary carcinoma	Miniature Dachshund	13	N	M
Tubulopapillary carcinoma	Miniature Dachshund	12	N	M
Tubulopapillary carcinoma	Miniature Dachshund	12	Y	M
Tubulopapillary carcinoma	Miniature Dachshund	12	N	M
Tubulopapillary carcinoma	Mixed	13	N	M

Abbreviations: M, metastasis; N, no; NM, non‐metastasis; Y, yes.

Furthermore, we used five previously established canine mammary tumour cell lines [CHMp (RRID:CVCL_L147), CIPp (RRID:CVCL_L149) and CTBp (RRID:CVCL_L151), which were derived from primary mammary lesions and CHMm (RRID:CVCL_L146) and CIPm (RRID:CVCL_L148), which were derived from metastatic lesions.] [17] in addition to the clinical samples, for qRT‐PCR validation.

After screening with only the clinical tissue samples, both clinical tissues and cell lines were grouped according to the metastatic status of the donor case rather than the anatomical origin of the sampled lesion for qRT‐PCR validation. Therefore, primary lesion‐derived materials from dogs with confirmed metastasis were included in the metastatic group.

On receipt at our laboratory, each tissue sample and each cell line was immersed in RNAlater and placed in a 4°C refrigerator overnight and then transferred to a −80°C freezer for storage until use in this study.

### 2.2 Cell Line Validation Statement

The CIPp, CIPm, CTBp, CHMm and CHMp cell lines were obtained from the University of Tokyo. Cell lines were confirmed to be uncontaminated and in the original report, the cell lines were characterised by light microscopy, electron microscopy and keratin/vimentin immunohistochemistry. Details on these cell lines can be found in past research [17].

### 2.3 Total RNA Extraction and Sequencing Analysis

Total RNA was extracted using a mirVana miRNA isolation kit (Thermo Fisher Scientific Inc.), following the manufacturer's instructions and the total RNA's concentration and quality were determined using a NanoDrop 2000c (Thermo Fisher Scientific) and the 2100 Bioanalyzer System (Agilent Technologies), separately. Samples with an RNA integrity number (RIN) exceeding eight were submitted for NGS analysis. Following RNA isolation and quality assurance, mRNA libraries were prepared and sequenced by Hokkaido System Science Co. Ltd. (Hokkaido, Japan). RNA libraries were constructed from 0.5 μg of total RNA using the TruSeq Small RNA Library Preparation Kit (Illumina, San Diego, CA, USA). The adapter sequences were as shown below. Forward adapter sequence: TGGAATTCTCGGGTGGCCAAGG Reverse adapter sequence: GATCGTCGGACTGTAGAACTCTGAAC. The libraries were subjected to 100‐bp paired‐end sequencing on an Illumina HiSeq 2500 System (Illumina).

### 2.4 RNA Sequencing Data Process and Differentially Expression Analysis

CLC Genomics Workbench 23.0.1 (CLC bio, Cambridge, MA, USA) was used to process and analyse the RNA sequencing data (with steps including quality control, annotation and normalisation of reads). The Illumina files (forward and reverse reads) were merged during import (Quality scores: NCBI/Sanger or Illumina Pipeline 1.8).

Differentially expressed mRNA and miRNA were selected by processing RNA‐seq data (GE, Gene level) with the Empirical Analysis of DEGs tool in CLC Genome Workbench 23.0.1. The miRNA‐seq data analyzed in the present study were generated from selected canine MGT samples reported in our previous study [11], whereas the mRNA‐seq data were generated from selected canine MGT samples reported in another previous study by our group [18]. These datasets were reanalyzed here to investigate candidate mRNA–miRNA relationships associated with primary canine MGTs from dogs with confirmed metastasis. The mRNA reads were annotated against the Ensembl canine database (ROS_Cfam_1.0.107) [19] and the miRNA reads were annotated by miRBase [20]. Differentially expressed genes and miRNAs were to be selected based on a false discovery rate *p* value (FDR *p* value) ≤ 0.05 and |log_2_FC| ≥ 1. Because transcriptome‐wide differential expression analysis involves multiple testing across a large number of transcripts, FDR‐adjusted *p* values were considered as a measure of statistical support after multiple‐testing correction [21]. The |log_2_FC| ≥ 1 threshold was used as a practical exploratory filter because statistical significance alone does not necessarily reflect the magnitude of expression change [22]. In our NGS dataset, transcripts showed a wide range of expression changes; therefore, this cutoff was used together with FDR ≤ 0.05 to reduce the candidate list and to focus on transcripts showing differences in expression that were both statistically significant and relatively large. This threshold was not intended to indicate biological significance by itself. For candidates that met the initial thresholds, prioritisation was not based on log_2_FC alone. Expression values across individual samples were also evaluated because RNA‐seq differential expression analyses can be influenced by outlying observations [23]. This step was used to evaluate whether the observed group difference showed a broadly interpretable expression pattern across samples or was skewed by a single or small number of outlying values. Predicted RIPPLY1‐targeting miRNAs were subsequently compared with the differentially expressed miRNAs identified in the NGS dataset to prioritise candidate miRNA–mRNA relationships for downstream validation. Log_2_FC was calculated for the expression value and mRNA expression value was normalised as transcripts per kilobase million (TPM) with miRNA expression value units shown as total counts. Differential expression is visualised by SRplot, an online platform for data analysis and visualisation [24].

### 2.5 Analysis of RIPPLY1 and miR‐187 Expression in Human Breast Cancer Database

Expression data for RIPPLY1 and miR‐187 were obtained from the Cancer Genome Atlas (TCGA) database and analysed using the UALCAN online platform for differential expression [25].

### 2.6 Quantification of mRNA and miRNA by qRT‐PCR

For mRNA, total RNA (25 ng/μL) was reversed transcribed to cDNA by using ReverTra Ace qPCR RT Master Mix with gDNA Remover (Toyobo) following the manufacturer's instructions. qRT‐PCR was performed in duplicate using a TaqMan Fast Advanced Master Mix kit and a StepOne Plus Real Time PCR system (Thermo Fisher Scientific). Expression levels were determined using the 2^−ΔΔCT^ method and GAPDH was used as a housekeeping gene in this study. The TaqMan Gene Expression Assay used in this study was GAPDH (ID:cf04419463_gH), RIPPLY1 (ID:cf02659795_g1).

For miRNA, total RNA (2 ng/μL) was reverse transcribed to cDNA using the TaqMan MicroRNA Reverse Transcription kit (Thermo Fisher Scientific) according to the manufacturer's protocol. qRT‐PCR was performed using a TaqMan Fast Advanced Master Mix kit and a StepOne Plus Real Time PCR system (Thermo Fisher Scientific). Expression levels were determined using the 2^−ΔΔCT^ method and RNU6B was used as a housekeeping gene in this study. The TaqMan MicroRNA assays used in this study and their IDs are as follows: RNU6B (ID: 001093), cfa‐miR‐187 (ID: 001193).

### 2.7 MiRNA Binding Site Prediction and PPI Network Analysis

The miRNA binding site was predicted by miRWalk [26] and RNAhybrid [27]. The RNAhybrid settings comprised one hit per target and an energy threshold of −20. To further evaluate canonical 3′‐UTR‐mediated regulation, cfa‐miR‐187 target genes were additionally predicted using TargetScan. Predicted targets were filtered based on‐site type (8mer/7mer‐m8) and cumulative weighted context++ score ≤ −0.3. The protein–protein interaction network (PPI network) was predicted by the STRING database [28] and visualised using Cytoscape (version 3.10.2) [29]. An MCODE algorithm was used within the Cytoscape application for cluster network analysis [30]. Pathway prediction visualised by SRplot [24].

### 2.8 Statistical Analysis

qRT‐PCR expression data were analysed using GraphPad Prism 10 (GraphPad Software Inc., San Diego, California) and also used to make the graph. The qPCR data were analysed by the Mann–Whitney U test. A *p* value < 0.05 was considered statistically significant. Correlation analysis was performed using Spearman's correlation coefficient and relative expression values of RIPPLY1 and miR‐187 used for correlation analysis were log‐transformed.

## 3 Results

### 3.1 Differentially Expressed mRNAs and miRNAs in Primary Canine MGT Tissue With or Without Metastasis in NGS Analysis

To identify differentially expressed miRNAs and mRNAs, we subjected primary canine MGT tissue samples from dogs with confirmed metastasis (*n* = 6) and without confirmed metastasis (*n* = 3) to NGS analysis.

**FIGURE 1:**
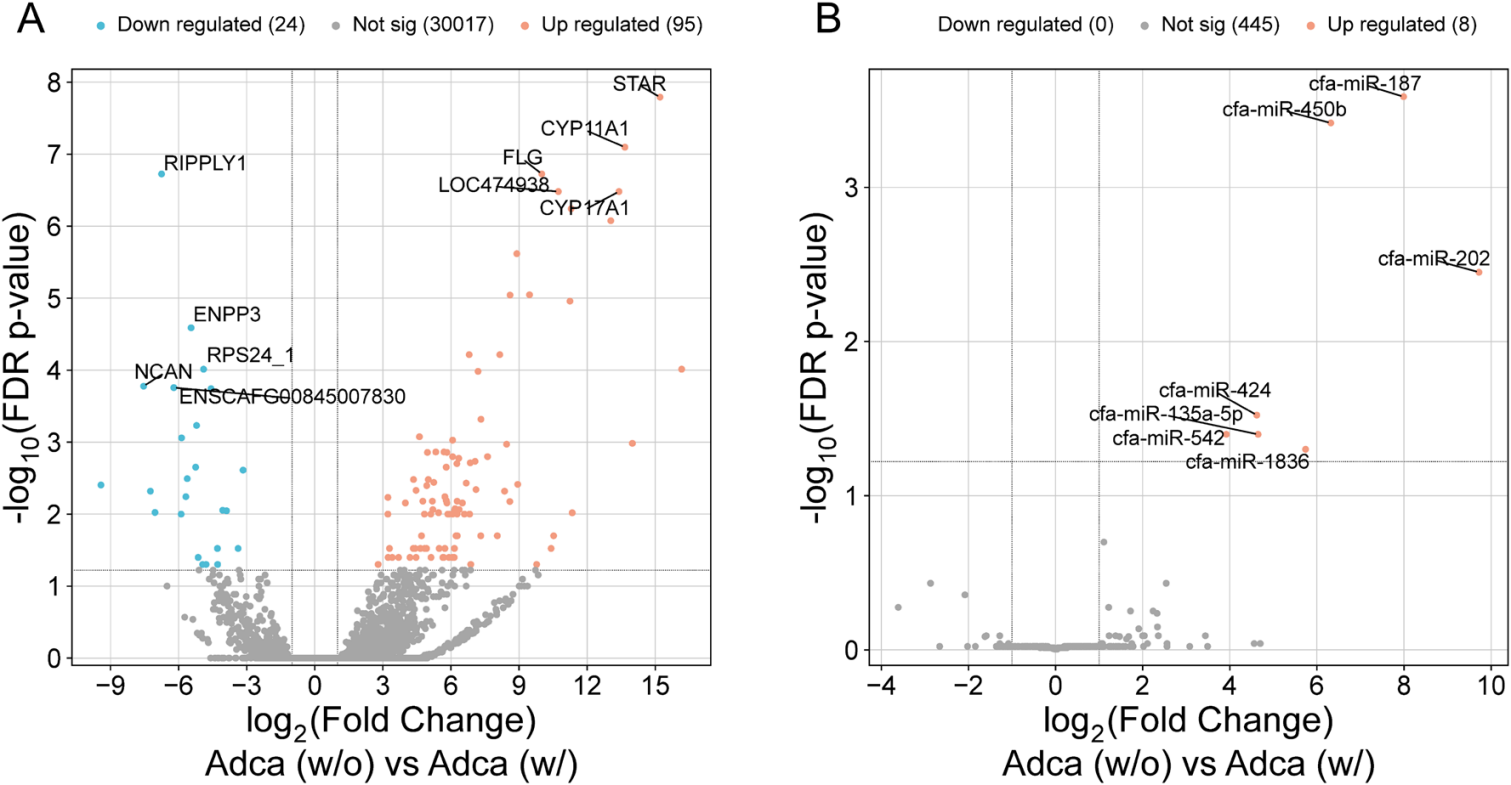
NGS analysis results are visualised in a volcano plot. (A) Differentially expressed mRNAs are visualised with red dots indicating upregulated candidates meeting the screening criteria (FDR *p*‐value ≤ 0.05 and |log_2_FC| ≥ 1) and blue dots indicating downregulated candidates meeting the same criteria. (B) Differentially expressed miRNAs are visualised with red dots indicating upregulated candidates meeting the screening criteria.

A total of 119 genes were differentially expressed, of which 95 were upregulated and 24 were downregulated (Table 2; Table S1). The results of this mRNA expression analysis have been visualised in a volcano plot (Figure 1A). A total of eight miRNAs were differentially expressed, all of which were upregulated. The results of the miRNA analysis are visualised in a volcano plot (Figure 1B, Table 3; Table S2).

**TABLE 2:** List of top 10 upregulated and 10 downregulated mRNA ranking by false discovery rate *p* value from NGS data.

Feature ID	FDR *p*‐value	Log_2_ fold change
STAR	1.61E‐08	15.21
CYP11A1	7.98E‐08	13.66
FLG	1.88E‐07	10
CYP17A1	3.30E‐07	13.4
LOC474938	3.30E‐07	10.73
CSN1S1	5.72E‐07	11.29
ENSCAFG00845014518	8.40E‐07	13.04
CYP21A2	2.41E‐06	8.9
OSGIN1_1	8.97E‐06	9.46
CSN2	9.05E‐06	8.6
RIPPLY1	1.88E‐07	−6.75
ENPP3	2.58E‐05	−5.45
RPS24_1	9.71E‐05	−4.9
NCAN	1.67E‐04	−7.54
ENSCAFG00845007830	1.75E‐04	−6.21
FBN2	1.81E‐04	−4.57
LRP2	5.87E‐04	−5.21
GFAP	8.73E‐04	−5.87
PTN	2.23E‐03	−5.25
SCGB1A1	2.45E‐03	−3.16

**TABLE 3:** Differentially expressed miRNAs in NGS analysis ranked by false discovery rate *p* value.

Feature ID	FDR *p*‐value	Log_2_ fold change
cfa‐miR‐187	2.58E‐04	7.99
cfa‐miR‐450b	3.82E‐04	6.32
cfa‐miR‐202	3.55E‐03	9.72
cfa‐miR‐424	0.03	4.62
cfa‐miR‐503	0.03	4.62
cfa‐miR‐135a‐5p	0.04	3.92
cfa‐miR‐542	0.04	4.65
cfa‐miR‐1836	0.05	5.74

Although several differentially expressed genes showed larger absolute log_2_FC values than RIPPLY1, candidate prioritisation for downstream validation was not based on log_2_FC alone. After applying the initial screening criteria of FDR ≤ 0.05 and |log_2_FC| ≥ 1, candidate genes were further evaluated according to FDR‐adjusted *p* value and sample‐level expression distribution. Some genes with very large mean log_2_FC values showed expression patterns skewed by a single outlier value or a small number of outlier values and were therefore considered unsuitable for downstream validation.

RIPPLY1 was prioritised as a downregulated mRNA candidate for exploratory investigation because it showed a strong measure of statistical support, a large absolute log_2_FC value and clearly decreased expression in primary tumour samples from dogs with confirmed metastasis. To provide a more detailed illustration of the distribution of values, sample‐level expression values of representative differentially expressed genes are shown in Table S1.

To prioritise a candidate miRNA related to RIPPLY1, predicted RIPPLY1‐targeting miRNAs were compared with the differentially expressed miRNAs identified in the NGS dataset. Among the overlapping candidates, cfa‐miR‐187 and cfa‐miR‐1836 were identified as possible RIPPLY1‐targeting miRNAs in the miRWalk database. cfa‐miR‐187 was prioritised for qRT‐PCR validation because it showed a stronger measure of statistical support, with the lowest FDR‐adjusted *p* value among the differentially expressed miRNAs, whereas cfa‐miR‐1836 showed weaker FDR support. Therefore, the RIPPLY1–cfa‐miR‐187 relationship was selected as the candidate pair for qRT‐PCR validation in an exploratory investigation aimed at hypothesis generation.

### 3.2 In Silico Prediction of a Candidate cfa‐miR‐187–RIPPLY1 Interaction

To explore a possible candidate relationship between a differentially expressed miRNA and RIPPLY1, miRNA target prediction tools were applied to identify a possible binding site.

**FIGURE 2:**
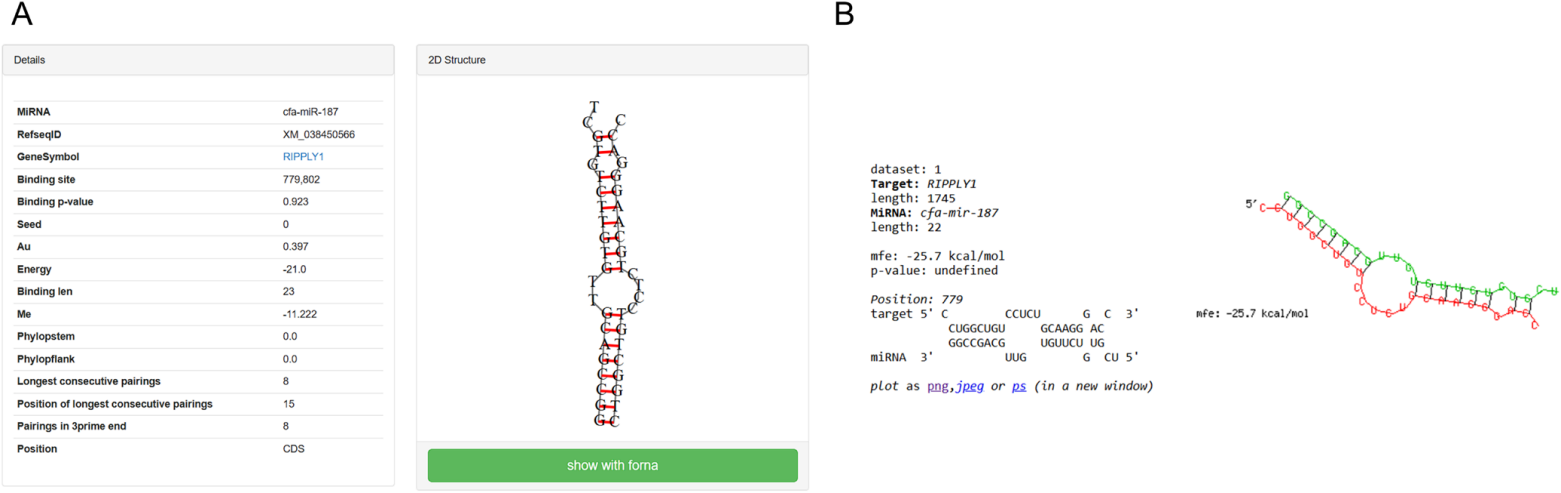
RIPPLY1 and miR‐187 binding site prediction. (A) Binding site prediction results with miRWalk. (B) Binding site analysis results from RNAhybrid.

Initially, the online database miRWalk was used and the putative binding site for miR‐187 was identified at nucleotide 779– 802 within the coding sequence (CDS) of RIPPLY1. This site exhibited a binding *p*‐value close to one (0.923) and predicted binding energy of −21 kcal/mol, which suggests a stable interaction (Figure 2A). Based on the miRWalk prediction model, this region is considered a predicted candidate binding site for miR‐187.

To substantiate this finding, we evaluated the binding site using RNAhybrid and found a similar binding site within the RIPPLY1 CDS, also around position 779 with a minimum free energy (MFE) of −25.7 kcal/mol, which suggests a thermodynamically favourable predicted interaction (Figure 2B). To place the predicted cfa‐miR‐187‐RIPPLY1 relationship in a broader context, we additionally performed canonical 3′‐UTR target prediction using TargetScan. Under the filtering criteria used in this study, 11 candidate mRNAs were identified as predicted canonical 3′‐UTR targets of cfa‐miR‐187 (Table S3). However, none of these predicted targets overlapped with the differentially expressed genes identified in our NGS dataset. Accordingly, the computationally predicted cfa‐miR‐187–RIPPLY1 interaction may be interpreted as a potentially hypothesis‐generating finding.

### 3.3 qRT‐PCR Validated RIPPLY1 and miR‐187 Relative Expression and Correlation in Clinical Tissue and Cell Lines

Based on the NGS‐based candidate prioritisation and in silico miRNA–mRNA prediction described above, RIPPLY1 and cfa‐miR‐187 were selected for qRT‐PCR validation. This validation was intended to confirm the differential expression patterns of these candidates in an exploratory investigation, rather than to establish a direct regulatory mechanism. To validate the NGS results, we targeted samples from a wider sample set comprising 13 non‐metastatic clinical tissue samples, 10 metastatic clinical tissue samples and 5 cell line samples classified into the metastatic group according to the metastatic status of the donor case for qRT‐PCR analysis to confirm expression levels for these two transcripts.

**FIGURE 3:**
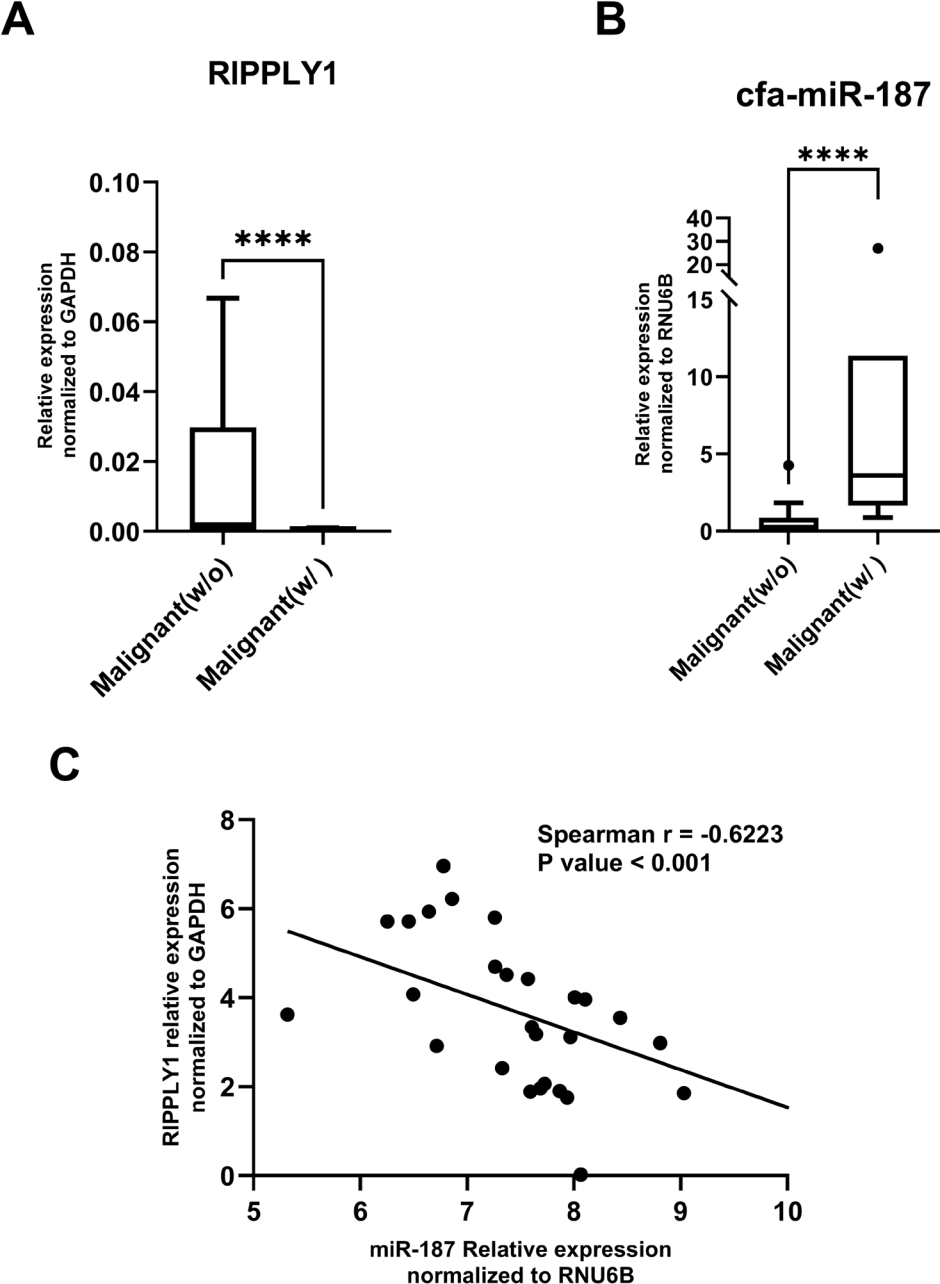
Relative expression of RIPPLY1 and miR‐187 in primary canine MGTs from dogs with or without confirmed metastasis, as validated by qRT‐PCR. (A) Relative expression of RIPPLY1. (B) Relative expression of miR‐187. (C) Correlation between RIPPLY1 and miR‐187 (Spearman's correlation coefficient). *p < 0.05, **p < 0.01, ***p < 0.001, ****p < 0.0001. Spearman r, Spearman correlation. Malignant (w/o), primary canine MGTs from dogs without confirmed metastasis. Malignant (w/), primary canine MGTs from dogs with confirmed metastasis.

In canine samples, RIPPLY1 expression was significantly lower in the primary MGT samples with confirmed metastasis than in those without confirmed metastasis (Figure 3A) and miR‐187 expression was significantly greater in the primary MGT samples with confirmed metastasis (Figure 3B). These results supported the differential expression pattern observed in the NGS analysis.

To further investigate the potential RIPPLY1‐miR‐187 interaction, we evaluated transcript expression using Spearman's rank correlation coefficient. We found that miR‐187 expression and RIPPLY1 expression were significantly negatively correlated (−0.6223 and *p* value < 0.001; Figure 3C) and this inverse association between cfa‐miR‐187 and RIPPLY1 expression may be regarded as a hypothesis‐generating finding based on our exploratory investigation using primary tumour samples from dogs with confirmed metastasis.

### 3.4 RIPPLY1 and miR‐187 Expression Level in Human Database

To investigate the extent to which the RIPPLY1 downregulation/miR‐187 upregulation pattern in canine MGT is reflected in human breast cancer, we analysed data from TCGA database using the UALCAN portal to determine relative RIPPLY1 and miR‐187 expression levels in normal, metastatic and non‐metastatic human breast cancer samples and survival analysis to evaluate the prognostic values of these two transcripts.

**FIGURE 4:**
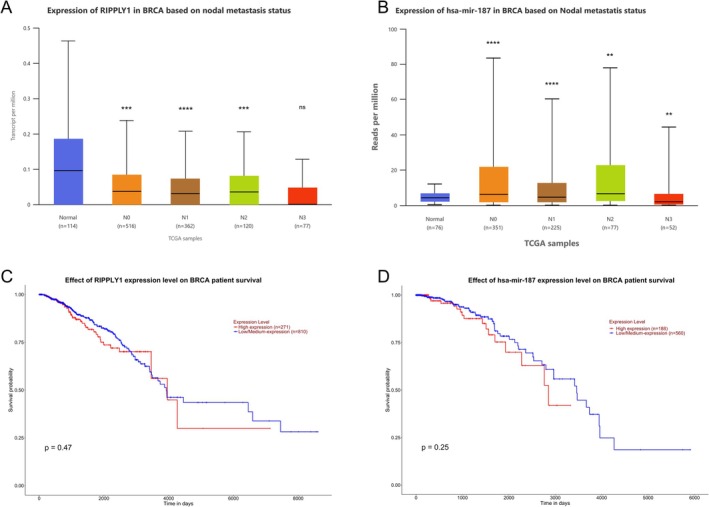
RIPPLY1 and miR‐187 expression levels in human breast cancer data from the TCGA database. (A) RIPPLY1 expression from normal tissue vs. tumour tissue (N0, N1, N2 and N3). (B) miR‐187 expression from normal tissue vs. tumour tissue. (C) Kaplan–Meier survival analysis of RIPPLY1. (D) Kaplan–Meier survival analysis of miR‐187. N0, No regional lymph node metastasis. N1, Metastases in 1 to 3 axillary lymph nodes. N2, Metastases in 4 to 9 axillary lymph nodes. N3, Metastases in 10 or more axillary lymph nodes. p, *p*‐value. *p < 0.05, **p < 0.01, ***p < 0.001, ****p < 0.0001.

In the human TCGA dataset, RIPPLY1 is significantly downregulated in tumour tissue relative to normal breast tissue; however, it showed no significant difference between non‐metastatic breast cancer cases (N0) and those with metastasis (N1, N2 or N3; Figure 4A; Table S4). Kaplan–Meier curve analysis indicated RIPPLY1 is not significantly associated with prognosis (Figure 4C).

miR‐187 was significantly upregulated in both non‐metastatic and metastatic breast cancer tissue versus normal; and in cases with N3 breast cancer cases compared with non‐metastatic breast cancer (N0 vs. N3; Figure 4B; Table S4). miR‐187 was not correlated with patient prognosis in Kaplan–Meier analysis (Figure 4D). Overall, these findings suggest that RIPPLY1 and miR‐187 may contribute to tumorigenesis in human breast cancer, although neither is related to prognosis in human breast cancer. These results suggest that, in humans, RIPPLY1 and miR‐187 are more closely associated with tumour versus normal differences than with a clear metastasis‐associated pattern. Therefore, the TCGA analysis should be interpreted as providing possible cross‐species contextual evidence for tumour biology or neoplasia, rather than as validation of a conserved metastasis‐specific role.

### 3.5 Exploratory Protein–Protein Interaction Network Analysis of RIPPLY1

To explore the possible biological context of RIPPLY1 in canine MGTs, we constructed a PPI network using STRING, followed by pathway and network analysis.

**FIGURE 5:**
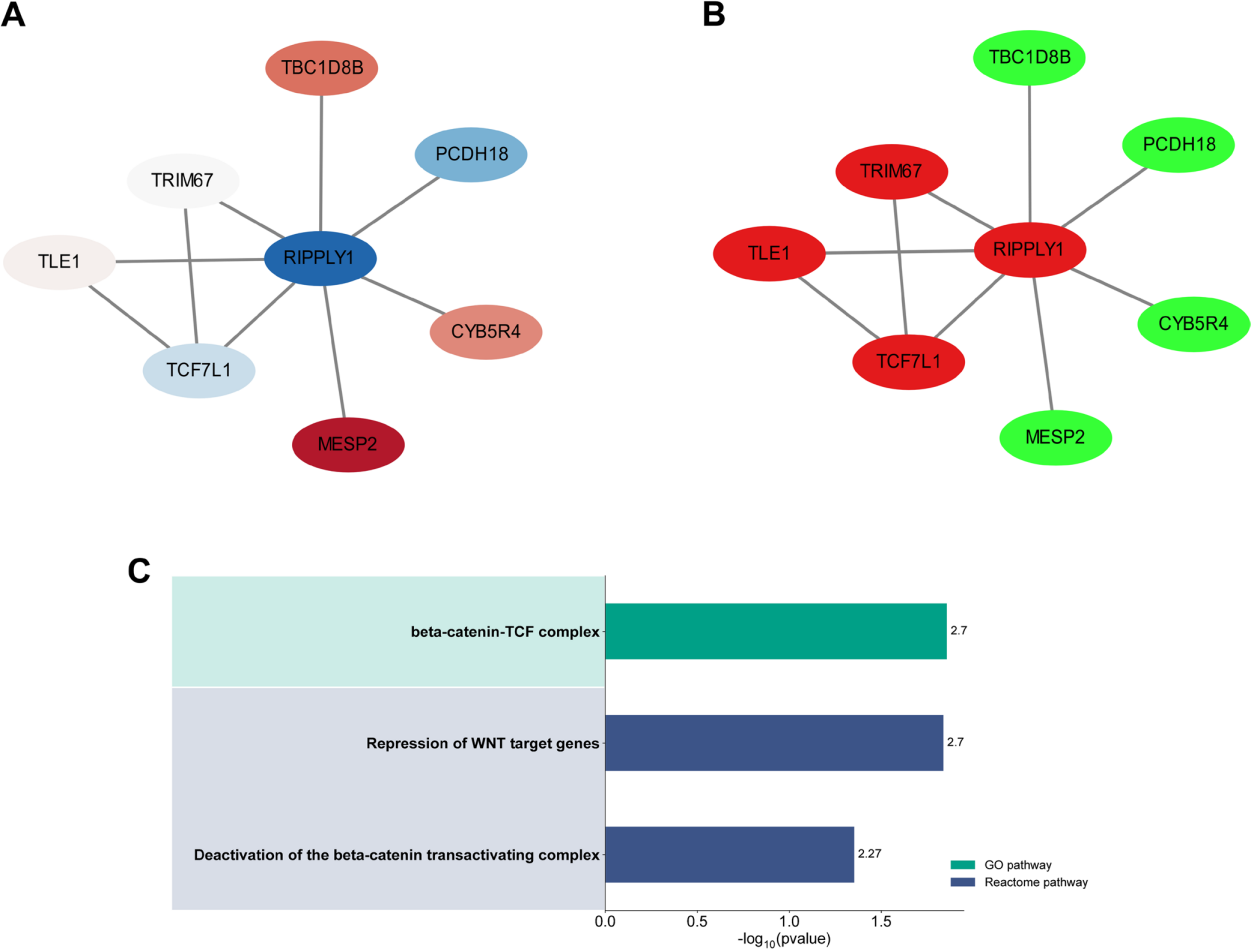
RIPPLY1's PPI network and pathway analysis results based on the STRING database and visualised by Cytoscape. (A) The PPI network related to RIPPLY1, with red indicating upregulation in NGS analysis data and blue indicating downregulation in NGS data. (B) MCODE algorithm analysis, with red genes representing a highly interconnected region in the PPI network. (C) Pathways related to the PPI network were analysed based on the STRING database and visualised by a bar chart.

In the generated PPI network, genes were colourized according to their fold change values derived from the NGS data (Figure 5A). Although most genes that interacted with RIPPLY1 were not significantly differentially expressed in our NGS analysis, the STRING‐based network may still provide preliminary information regarding the possible biological context of RIPPLY1.

To investigate interconnected regions in the PPI network, we applied the MCODE algorithm. The most highly connected module (Mscore = 3.33) was identified with RIPPLY1 (Figure 5B) included as one of the core genes. According to the network analyser, RIPPLY1 exhibited a degree centrality of three and these preliminary findings suggest that RIPPLY1 may occupy an interconnected position within this network.

Functional enrichment analysis of the network revealed a significant association with the beta‐catenin‐TCF complex, which is the main component of the Wnt/β‐catenin signalling pathway (Figure 5C). These results suggest a possible pathway context for RIPPLY1‐associated proteins. However, because STRING analysis is computational, these findings should be interpreted as preliminary pathway information rather than evidence that RIPPLY1 directly regulates Wnt/β‐catenin signalling in canine MGTs.

## 4 Discussion

In this study, we aimed to identify differentially expressed transcripts (mRNA and miRNA) in canine primary MGTs with or without confirmed metastasis. NGS profiling of gene expression revealed that RIPPLY1 was significantly downregulated, whereas miR‐187 was upregulated in primary MGTs with confirmed metastasis. This expression pattern was further validated in qRT‐PCR, based on which we found a significantly negative correlation between miR‐187 and RIPPLY1 expression levels. In silico analyses also predicted a possible binding site between miR‐187 and RIPPLY1. However, these findings are based on expression association and computational prediction. Therefore, the candidate RIPPLY1‐miR‐187 relationship should be interpreted as a hypothesis‐generating finding rather than as evidence of direct post‐transcriptional regulation.

The use of bulk primary tumour tissues also affects the interpretation of the present findings. The confirmed‐metastasis group in this study consisted of samples from primary mammary tumours from dogs with confirmed metastasis, rather than metastatic lesions themselves. Thus, the observed RIPPLY1 and cfa‐miR‐187 expression changes should be interpreted as candidate molecular features associated with primary tumours from dogs with confirmed metastasis, not as features proven in metastatic tumour cells. Because canine MGTs are heterogeneous [15, 16] and no cell sorting or microdissection was performed, the expression profiles may reflect signals from both neoplastic epithelial cells and non‐neoplastic components of the tumour microenvironment, such as immune cells and fibroblasts [31]. Future studies using matched metastatic lesions and single‐cell RNA sequencing will be required to clarify the cellular origin and biological relevance of the candidate RIPPLY1–cfa‐miR‐187 relationship.

Previous studies have reported that miR‐187 is involved in tumour cell proliferation and metastasis progression in several human cancers, including human breast cancer [32], gastric cancer [33] and clear cell renal cell carcinoma [34]. These reports support the possible relevance of miR‐187 to cancer biology. However, they do not establish a functional role for cfa‐miR‐187 in canine MGTs and direct experimental validation is required. On the other hand, RIPPLY1 is a member of the Ripply family of transcriptional corepressors and has mainly been studied in developmental processes. Previous studies showed that Ripply family proteins are involved in somite segmentation and can convert T‐box transcription factors from activators to repressors [35, 36]. RIPPLY1 expression has also been reported in MDCK organoid models, suggesting a possible role in epithelial morphogenesis [37]. The role of RIPPLY1 in cancer remains poorly characterised. However, a recent study suggested that RIPPLY1 may suppress cancer cell stemness in CTNNB1‐mutated hepatocellular carcinoma [38]. These findings suggest that RIPPLY1 may have biological relevance beyond embryonic development and epithelial organisation. Nevertheless, the role of this gene in canine MGTs remains to be clearly elucidated and the present findings should be considered as preliminary.

In our STRING‐based PPI analysis, RIPPLY1‐associated proteins were linked to the beta‐catenin/TCF complex. This result may provide a possible biological context for RIPPLY1, because Wnt/β‐catenin signalling is frequently involved in cancer progression [39, 40, 41]. However, because STRING analysis is based on computationally predicted and curated protein–protein associations, this result should be interpreted as preliminary pathway information, not as evidence that RIPPLY1 directly regulates Wnt/β‐catenin signalling or metastatic progression in canine MGTs.

miRNAs typically regulate gene expression by binding to the 3′ untranslated region (3′UTR). However, our binding site prediction suggested a possible cfa‐miR‐187 binding site within the RIPPLY1 CDS region rather than the 3′UTR region. Although less common, CDS targeting by miRNAs has been previously reported and is regarded as a non‐canonical miRNA‐mediated regulation [42, 43, 44, 45]. The possibility of non‐canonical miRNA‐regulated mRNA to suppress tumour cell growth has been explored in previous studies [45, 46, 47]. Although our miRWalk and RNAhybrid analyses suggested a possible interaction between cfa‐miR‐187 and RIPPLY1 within the coding region, canonical 3′‐UTR‐mediated regulation is more commonly associated with miRNA function. Additional TargetScan analysis identified several predicted canonical 3′‐UTR targets of cfa‐miR‐187, although none overlapped with the differentially expressed genes in our dataset. Therefore, the proposed cfa‐miR‐187‐RIPPLY1 interaction should be considered a computationally predicted and hypothesis‐generating finding and direct functional validation will be required in future studies.

To investigate the potential relevance of our canine MGT findings to human breast cancer, we analysed RIPPLY1 and miR‐187 expression using the TCGA database. In the human dataset, both transcripts showed expression differences mainly between normal breast tissue and tumour tissue, but not a clear metastasis‐associated pattern. Thus, the TCGA results support the possible relevance of RIPPLY1 and miR‐187 to human breast tumour biology, rather than validating a conserved metastasis‐specific role. This discrepancy may reflect the molecular heterogeneity of human breast cancer and the limitations of broad TCGA comparisons based on tumour‐normal or nodal status [48]. Therefore, future subtype‐based comparative studies will be required to clarify whether the RIPPLY1–cfa‐miR‐187 candidate relationship is conserved in specific tumour contexts [49, 50].

Although RIPPLY1 and cfa‐miR‐187 expression levels were significantly negatively correlated, this association does not confirm direct regulation. Because this study used bulk primary mammary tumour tissues rather than metastatic lesions or isolated tumour cells, the observed expression differences should be interpreted as candidate molecular features associated with primary tumours from dogs with confirmed metastasis. Further functional studies using luciferase reporter assays, miRNA mimic/inhibitor experiments, protein‐level validation and matched primary/metastatic tumour samples will be required to clarify the cellular origin and biological relevance of the candidate RIPPLY1–cfa‐miR‐187 relationship.

In conclusion, this study identified RIPPLY1 and cfa‐miR‐187 as candidate transcripts differentially expressed in primary mammary tumour samples from dogs with or without confirmed metastasis. Their inverse expression pattern and in silico binding predictions suggest a possible RIPPLY1–cfa‐miR‐187 candidate relationship. However, this relationship remains hypothesis‐generating and requires functional validation using appropriate experimental models and matched primary/metastatic tumour samples. TCGA analysis supported the possible relevance of RIPPLY1 and miR‐187 to human breast tumour biology, but did not indicate a conserved metastasis‐specific pattern.

## Author Contributions

**Shaohsu Wang:** conceptualization, methodology, validation, formal analysis, investigation, resources, visualisation, writing – original draft, writing – review and editing. **Masashi Takahashi:** conceptualization, methodology, resources, writing – review and editing, supervision. **Hui‐Wen Chen:** methodology, validation, formal analysis, visualisation. **Nobuhiro Nozaki:** validation, formal analysis, resources, visualisation. **Mohammad Arif:** validation, formal analysis, resources, visualisation. **Yutaro Ide:** validation, formal analysis, resources. **Yoshiyuki Akiyama:** validation, formal analysis, resources. **Sirazul Islam:** validation, formal analysis, resources. **Tatsuro Hifumi:** formal analysis, resources. **Shinji Hirano:** formal analysis, resources. **Noriaki Miyoshi:** formal analysis, resources. **Osamu Yamato:** formal analysis, resources. **Daiki Kato:** formal analysis, resources. **Takayuki Nakagawa:** formal analysis, resources. **Naoki Miura:** conceptualization, methodology, writing – review and editing, supervision, project administration, funding acquisition.

## Funding

This work was supported by the Japan Society for the Promotion of Science (JSPS) KAKENHI [grant numbers 21H02366 and 20K21375].

## Disclosure

Cell lines were used in this study. We used canine‐origin cell lines CIPp, CIPm, CTBp, CHMm and CHMp in this study.

## Ethics Statement

This article does not contain any studies involving human participants. Informed consent was obtained from the owner of each dog from which tissues were collected. This study was approved by the Ethics Committee at KUVTH (KVH220001 and KVH240013). All human data used were obtained from publicly available TCGA datasets, with no requirement for additional ethical approval.

## Conflicts of Interest

The authors declare no conflicts of interest.

## Supporting information


**Table S1:** A total of 119 significantly DEGs analysed by NGS.

**Table S2:** A total of 8 significantly differentially expressed miRNA analysed by NGS.

**Table S3:** Predicted canonical 3′‐UTR targets of cfa‐miR‐187.

**Table S4:** TCGA statistical data.

## Data Availability

Sequence reads were submitted to the SRA (www.ncbi.nlm.nih.gov/sra) under the Bioproject accession number PRJNA716131 and PRJNA1478363.
